# Supplementary material for: Fabrication and Evaluation of Filtration Membranes from Industrial Polymer Waste
Source: Membranes (Basel). 2023 Apr 19;13(4):445. doi: 10.3390/membranes13040445 (PMC10143593; doi:10.3390/membranes13040445)
Supplement: Supplementary file 1 [file membranes-13-00445-s001.zip › membranes-2297992-supplementary.pdf]

Supplementary Information (SI)

Fabrication and evaluation of filtration membranes from industrial polymer waste

Saleheen Bano [1,2], Mukesh Pednekar [2,3,4], Saranya Rameshkumar [1,2], Dipu Borah [1,2], Michael A. Morris[1,2,*], Ramesh Babu Padamati [1,2,3,*] and Niamh Cronly[1]

[1] School of Chemistry, CRANN, Trinity College Dublin, Dublin 2, Ireland.
[2] AMBER, SFI Research Centre for Advanced Materials and BioEngineering Research, Ireland
[3] School of Physics, CRANN, Trinity College Dublin, Dublin 2, Ireland
[4] Dairy Processing Technology Centre (DPTC), Ireland
* Correspondence: babup@tcd.ie (R.B) ; morrism2@tcd.ie (M.A.M)

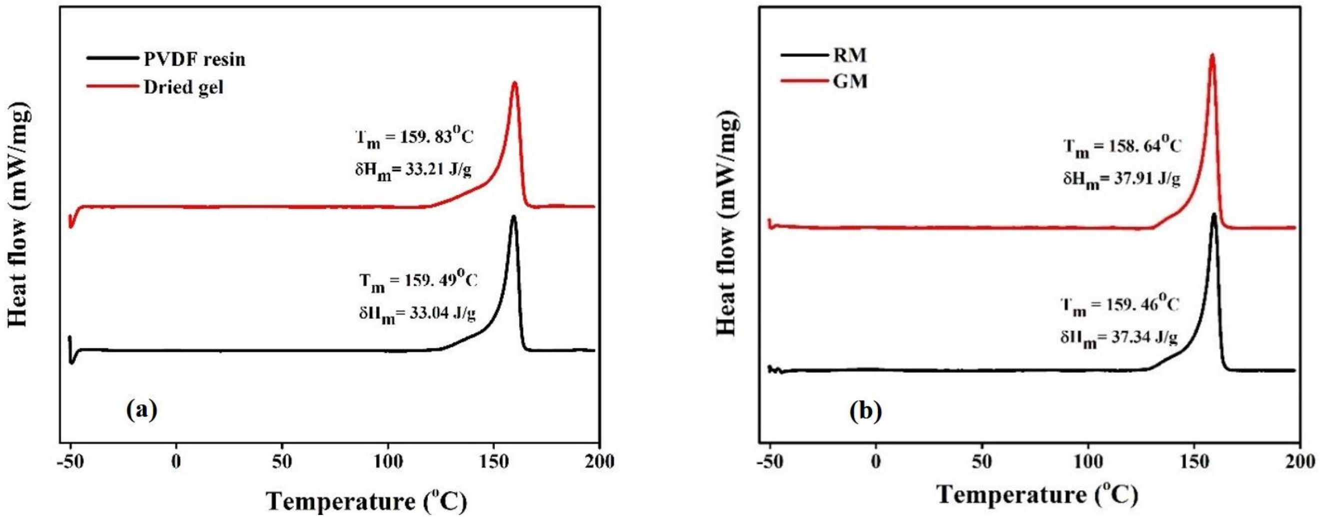

Figure S1. DSC scans of (a) gel powder and PVDF resin, (b) gel membrane (GM) and resin membrane (RM)

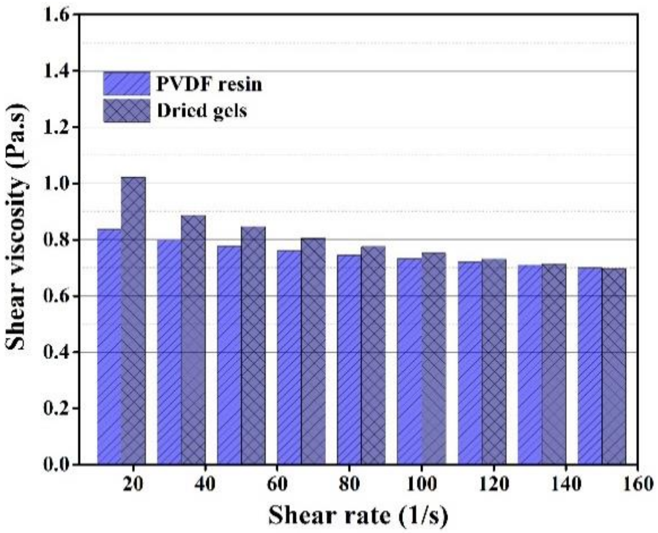

Figure S2. Shear viscosity data for dried gel powders and PVDF resins

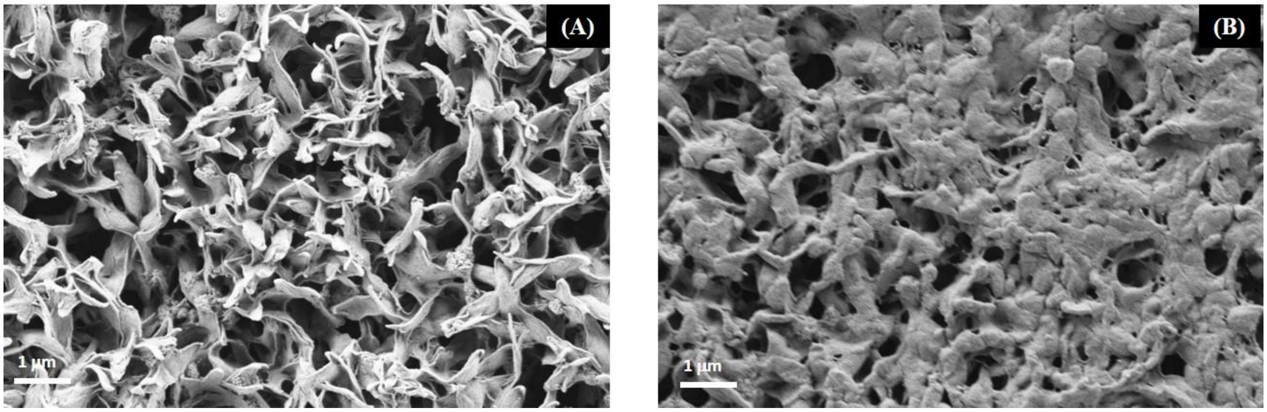

Figure S3. Surface morphology of (A) Fresh GM membrane and (B) fouled membrane after cleaning at the end of 2nd cycle of cross-flow filtration.

Table S1. Comparison of relevant data of the present study with previously reported data for PVDF based membranes prepared via phase inversion

Membrane	Solvent type	Pore size (μm)	Porosity (%)	PWP ($L/m^2.h$)	Retention (%)	Potential Application	Ref.
**PVDF- GM**	Acetone	0.19	66.7	478	26.8 [a]	MF	*Present study*
**PVDF ⁎**	DMA / acetone	0.25	35	430	-	UF	*(13)*
**PVDF⁎**	TEP	0.26<br>0.11	-	1860<br>120	12 [a]<br>97 [a]	MF-UF	*(44)*
**PVDF ⁎⁎**	TEP	0.14<br>0.17	86.5<br>82.8	290<br>1100	-	UF- MF	*(46)*
**PVDF ⁎⁎**	DMF<br>DMAc<br>DMAc/TEP<br>DMF/ TEP	-	32.2<br>33.7<br>44.2<br>8.2	99.6<br>87.7<br>89.6<br>35.2	-	MF	*(56)*
**PVDF ⁎⁎**	DCAC		78.7	258	18.4 [a]	MBR	*(41)*
**PVDF / 0.5GO-APTS**	DMF	0.26	57.2	6.2	99.9 [b]	AGDM	*(48)*
**PVDF / 0.005 nano-ZnO**	DMAc	0.08	75.1	452.1	29 [a]	MF	*(52)*
**PVDF (GVWP, Millipore)**	-	0.22	-	655	-	MF	*(57)*

⁎ Neat membrane without pore former; ⁎⁎ Neat membrane with pore former; a with respect to BSA; b with respect to mineral salt
